# Nonoptimal Virological Response Among Adults Living With HIV After Initiation of Combined Antiretroviral Therapy at a Tertiary Hospital in Oman: A Retrospective Cohort Study, 1992–2024

**DOI:** 10.1155/tswj/8552128

**Published:** 2026-10-21

**Authors:** Zainab M. Al-Zadjali, Faryal Khamis, Sanjay Jaju, Amal Saki Malehi, Sulaiman Dawood Al Sabei, Ruhina Aimaq, Jalila Al-Naamani, Ibtisam Khalifa Al-Maskari, Zakariya Al-Balushi, Saif Al-Abri, Alanood Al-Mandhari, Usama Al-Amri, Laila S. Al-Saadi, Yahya M. Al-Farsi

**Affiliations:** ^1^ Department of Family Medicine & Public Health College of Medicine & Health Sciences Sultan Qaboos University Muscat Oman squ.edu.om; ^2^ Department of Infectious Diseases Royal Hospital Ministry of Health Muscat Oman behdasht.gov.ir; ^3^ Department of Fundamentals and Administration College of Nursing Sultan Qaboos University Muscat Oman squ.edu.om

**Keywords:** cohort study, combined antiretroviral therapy, HIV, Oman, viral suppression, virological response

## Abstract

**Background:**

Combined antiretroviral therapy (cART) has transformed HIV infection from a life‐threatening condition into a manageable chronic disease by reducing viral replication, preserving immune function, and lowering HIV‐related morbidity and mortality. Nevertheless, achieving and maintaining viral suppression remains an important challenge. Evidence regarding factors associated with nonoptimal virological response among people living with HIV in Oman remains limited.

**Aim:**

This study investigated factors associated with nonoptimal virological response among adults living with HIV who initiated cART and received follow‐up at the Royal Hospital, Oman, between 1992 and 2024.

**Methods:**

A retrospective cohort study was undertaken at the Royal Hospital, Muscat, Oman, the national tertiary referral center for HIV care. The analysis included 549 adults aged ≥ 18 years who initiated cART and had the laboratory and clinical information required for the study. Participants were eligible if they had two consecutive HIV RNA measurements, both obtained at least 6 months after cART initiation. Optimal viral suppression was defined as HIV RNA ≤ 20 copies/mL, whereas nonoptimal virological response was classified only when both consecutive HIV RNA measurements were > 20 copies/mL, based on the laboratory and clinical monitoring threshold used at the Royal Hospital and Ministry of Health guidance. Participants with only one eligible measurement or discordant measurements were not classified as having a nonoptimal virological response. Clinical information was retrospectively retrieved from electronic medical records. Data were analyzed using IBM SPSS Statistics Version 28. Multivariable Poisson regression with robust variance was used to estimate adjusted risk ratios (aRRs) and corresponding 95% confidence intervals (CIs). Statistical significance was considered at *p* ≤ 0.05.

**Results:**

Of the 549 participants, 321 (58.5%) had HIV RNA ≤ 20 copies/mL and 228 (41.5%) had HIV RNA > 20 copies/mL. Compared with individuals diagnosed in 2020 or later, the risk of nonoptimal virological response was higher among those diagnosed in 2008 or earlier (aRR: 2.166, 95% CI: 1.160–4.043) and those diagnosed between 2009 and 2014 (aRR: 2.273, 95% CI: 1.258–4.107). Male participants had a higher risk than female (aRR: 2.154, 95% CI: 1.172–3.960). Diagnosis through antenatal care was also associated with nonoptimal virological response (aRR: 3.662, 95% CI: 1.263–10.618), although the estimate should be interpreted cautiously because of the small number of participants in this category. WHO clinical Stage 3 was associated with a lower estimated risk compared with Stage 4, although the association was not statistically significant (aRR: 0.592, 95% CI: 0.349–1.005; *p* = 0.052). Participants with hemoglobin levels ≤ 10 g/dL also had an increased risk of nonoptimal virological response (aRR: 1.676, 95% CI: 1.020–2.753).

**Conclusion:**

Five sociodemographic and clinical factors were independently associated with detectable HIV RNA among adults receiving cART in Oman: sex, period of HIV diagnosis, antenatal testing, WHO clinical stage, and hemoglobin concentration. These findings support continued efforts to identify patients who may require closer clinical monitoring and individualized support to achieve and maintain viral suppression.

## 1. Introduction

Over the past few decades, substantial progress has been made in the global response to the human immunodeficiency virus (HIV) or acquired immune deficiency syndrome (AIDS) epidemic [1]. However, HIV remains a major public health challenge, particularly in regions with fragile health systems and persistent stigma [2–4]. The introduction of combined antiretroviral therapy (cART) has significantly improved the prognosis of people living with HIV (PLWH) by suppressing viral replication, restoring immune function, and reducing HIV‐related morbidity and mortality [4–7]. The cART involves a regimen of three or more antiretroviral drugs aimed at reducing the viral load in the bloodstream [8]. Despite the global advance of treatment, new infections and deaths remain high. In 2025, an estimated 40.9 million people (37.1–45.7 million) were living with HIV globally, with 1.2 million (980,000–1.6 million) new infections and approximately 570,000 (440,000–740,000) AIDS‐related deaths [9]. About 32.1 million (28.2–33.4 million) people were receiving antiretroviral therapy. Since the start of the epidemic, 83.5 million (67.0–106.3 million) people have acquired HIV, and 43.2 million (36.8–52.3 million) have died from AIDS‐related illnesses [9].

Although there has been an overall decline in HIV incidence and AIDS‐related mortality, this trend is not consistent across all regions [1]. Notably, the Middle East and North Africa (MENA) region experienced a 116% increase in new HIV infections between 2010 and 2023 [10]. Within the MENA region, the Gulf Cooperation Council (GCC) countries, including Oman, face unique challenges. Conservative sociocultural and religious norms often restrict open discussions about HIV and sexual health, shaping public attitudes and contributing to stigma [10]. As of 2021, more than 42,000 individuals were living with HIV in the GCC, and incidence rates have been rising steadily since 1990 [10].

The benefits of cART are well established. Individuals who know their HIV status, adhere to treatment, and maintain viral suppression can live long, healthy lives and avoid transmitting the virus sexually, a principle known as “undetectable = untransmittable” (U=U) [11]. Sustained viral suppression is an important indicator of treatment response and is commonly defined using HIV RNA thresholds such as < 50, < 20, or < 5 copies/mL, depending on national guidelines and assay sensitivity [12–15]. In this study, virological response was assessed using HIV RNA measurements, with viral suppression defined as HIV RNA ≤ 20 copies/mL and nonoptimal virological response defined as HIV RNA > 20 copies/mL. Viral suppression reflects the effectiveness of antiretroviral treatment, whereas adherence is one of several factors that may influence virological response. High adherence is important for achieving and maintaining viral suppression [13, 14]. Studies show that viral suppression can reduce transmission risk by up to 95% among serodiscordant couples [5, 6], whereas individuals who do not receive cART have a nearly fivefold increased risk of death [16]. Despite this, many regions continue to struggle with nonoptimal adherence. In sub‐Saharan Africa, the average adherence rate is just 72.9%, and a regional meta‐analysis reported a wide range from 32.9% to 94%, underscoring the need for context‐specific solutions [4].

In the GCC region, similar challenges persist. Viral suppression and treatment coverage vary substantially across countries, reflecting disparities in care engagement [13, 17, 18]. Recent data show that awareness of HIV status among PLWH ranged from 66% in Qatar to 94% in Bahrain, whereas the proportion on cART varied from 58% in Qatar to 93% in Kuwait [17, 19]. Viral suppression rates among those on treatment also varied, with Kuwait at 92%, Saudi Arabia at 90%, Oman at 58%, and Bahrain at 55% [17]. These disparities highlight ongoing gaps in achieving the UNAIDS 95–95–95 targets [20].

In Oman, expansion of HIV treatment services has contributed to improvements in HIV care and reductions in community viral burden [2, 3]. However, determinants of nonoptimal virological response, assessed using routine laboratory viral load measurements, remain insufficiently investigated in Oman. Additionally, HIV‐related stigma continues to be a significant barrier to engaging in care [19]. Oman′s Health Vision 2050 emphasizes translating evidence into policy and practice to improve health outcomes [21]. To our knowledge, this is the first long‐term retrospective cohort study in Oman to evaluate factors associated with nonoptimal virological response among adults living with HIV who initiated cART, using laboratory‐collected clinical data over 32 years (1992–2024). The present study therefore examined factors associated with nonoptimal virological response among adults living with HIV who initiated cART and received care at the Royal Hospital, Oman, between 1992 and 2024. The primary outcome was defined using routine HIV RNA measurements rather than as a direct measure of treatment adherence or confirmed virological failure.

Importantly, the present analysis uses the same underlying cohort of adults living with HIV that has been described previously in our study of late HIV diagnosis [22]. However, the two studies address different research questions and outcomes. The previous study investigated factors associated with late HIV diagnosis, whereas the current study examines factors associated with virological response following cART initiation.

## 2. Materials and Methods

### 2.1. Study Design

To our knowledge, this is the first long‐term retrospective cohort study in Oman to evaluate factors associated with nonoptimal virological response among adults living with HIV who initiated cART, using routinely collected clinical and laboratory data over a 32‐year period (1992–2024). Although a previous study from the same cohort described the study design as ambidirectional ([22]), the present study is described as a retrospective cohort because the analysis was based on routinely collected clinical and laboratory records. For the present analysis, follow‐up for the virological outcome began at cART initiation, and outcome assessment required two consecutive eligible HIV RNA measurements obtained at least 6 months after cART initiation. Follow‐up for the virological outcome began at cART initiation. Outcome assessment required two consecutive eligible HIV RNA measurements, both obtained at least 6 months after cART initiation. Follow‐up continued through the third eligible HIV RNA measurement, with the study observation period ending on December 31, 2024. The present study therefore examined factors associated with nonoptimal virological response among adults living with HIV who initiated cART and received care at the Royal Hospital, Oman, between 1992 and 2024.

### 2.2. Study Setting

The study was conducted at the Royal Hospital, Muscat, which serves as the national tertiary referral center for HIV care in Oman. The hospital provides specialist management and long‐term follow‐up for adults living with HIV referred from different regions of the country. HIV treatment, laboratory monitoring, and clinical follow‐up are provided through specialized services involving infectious disease physicians and other healthcare professionals [23–25].

### 2.3. Characteristics of Participants: Inclusion and Exclusion Criteria

The study included adults aged ≥ 18 years living with HIV who initiated cART at the Royal Hospital between January 1, 1992, and December 31, 2024. Participants were eligible if they had two consecutive eligible HIV RNA measurements, both obtained at least 6 months after cART initiation, together with documented baseline hemoglobin data, according to the Royal Hospital HIV clinical monitoring guideline and Ministry of Health (MOH) guidance [14]. Participants with incomplete clinical or laboratory data required for the analysis were excluded.

### 2.4. Sample Size Calculation

All records meeting the predefined eligibility criteria during the study period were included; therefore, the final analysis represented a census of eligible records rather than a sample selected from the cohort. An a priori sample size calculation was nevertheless performed to assess whether the available number of participants was adequate for the planned analysis. Using OpenEpi Version 3.01, the calculation assumed a two‐sided test, 95% confidence level, 80% power, and 5% significance level [26, 27]. Estimates from a previously published HIV cohort were used to inform the calculation [2, 3]. The estimated minimum number required was 224 participants, increasing to 246 after allowing for 10% potential missing data. The final cohort of 549 eligible participants therefore exceeded the estimated minimum.

### 2.5. Sampling Method

No probability or nonprobability sampling method was used. All individuals who met the eligibility criteria during the defined study period were included. Accordingly, the study represents a census of eligible records available from the Royal Hospital HIV clinical database [28].

### 2.6. Data Source and Data Collection Tools

The data extraction sheet was developed based on the relevant literature and clinical information routinely collected for HIV care at the Royal Hospital, the national tertiary referral center for HIV management. The extraction sheet was reviewed by two HIV clinical experts to ensure content validity, completeness, and relevance to the study objectives. The sheet included sections covering sociodemographic characteristics, medical history, clinical characteristics, laboratory parameters, treatment‐related information, and study outcomes.

Eligible patients′ clinical records were obtained from the Royal Hospital′s Al‐Shifa 3+ electronic medical record system and extracted using a customized Excel‐based data extraction checklist. Data collection covered the study period from January 1, 1992, to December 31, 2024, and was conducted in accordance with approved ethical standards for human subjects′ research.

### 2.7. Study Variables

This was an observational cohort study and did not involve any intervention, participant interviews, or direct physical contact with patients. Data were retrospectively extracted from the Al‐Shifa 3+ electronic medical record system at the Royal Hospital. The collected variables included sociodemographic, clinical, laboratory, and treatment‐related characteristics. Sociodemographic variables included age at HIV diagnosis, sex, and marital status. Clinical variables included mode of HIV transmission, WHO clinical stage, comorbidities, and coinfections, including tuberculosis and viral hepatitis. Laboratory variables included baseline complete blood count (CBC) parameters, baseline CD4 cell count, and HIV viral load measurements at diagnosis and during follow‐up. Hemoglobin was categorized as ≤ 10 g/dL and > 10 g/dL.

A hemoglobin concentration of ≤10 g/dL was used as the analytical cutoff based on the clinical guidance used by the HIV clinic team at the study site and was also supported by previous studies that used a hemoglobin threshold of 10 g/dL [29, 30]. In this cohort, 128 participants (23.3%) had hemoglobin concentrations ≤10 g/dL, including eight participants (1.5%) with exactly 10.0 g/dL and 120 participants with concentrations below 10 g/dL. The primary outcome was nonoptimal virological response, defined as failure to achieve viral suppression, operationalized as HIV RNA > 20 copies/mL [14]. Viral load measurements recorded in Al‐Shifa 3+ were assessed after cART initiation, using follow‐up HIV RNA measurements obtained at least 6 months after starting cART; for the present analysis, eligibility required two consecutive eligible HIV RNA measurements, both obtained at least 6 months after cART initiation, according to routine clinical monitoring practice and applicable MOH guidance. The 6‐month interval was selected to allow sufficient time after cART initiation for virological response to be assessed during routine clinical follow‐up and to avoid relying on very early viral‐load measurements obtained shortly after treatment initiation.

### 2.8. Outcome Definition

The primary outcome was nonoptimal virological response after initiation of cART. For this study, HIV RNA ≤ 20 copies/mL was considered viral suppression, consistent with the laboratory reporting threshold and the HIV monitoring practices used at the Royal Hospital [14]. HIV RNA > 20 copies/mL was categorized as nonoptimal virological response.

For outcome classification, eligible HIV RNA measurements were defined as measurements obtained at least 6 months after cART initiation. Participants were required to have two consecutive eligible HIV RNA measurements, both obtained at least 6 months after cART initiation. HIV RNA ≤ 20 copies/mL was considered virological suppression, whereas HIV RNA > 20 copies/mL was classified as nonoptimal virological response. Participants were classified as having a nonoptimal virological response only when both consecutive eligible HIV RNA measurements were > 20 copies/mL. Participants with only one eligible measurement or discordant measurements (one > 20 copies/mL and one ≤ 20 copies/mL) were not classified as having a nonoptimal virological response. The outcome was interpreted as detectable viraemia rather than confirmed virological failure.

The 6‐month interval was selected to allow sufficient time after cART initiation for virological response to be assessed during routine clinical follow‐up, consistent with evidence evaluating virological suppression at 6 months after initiation of antiretroviral therapy [13, 14, 18].

This approach was selected to capture virological response during routine clinical monitoring. The outcome definition should be interpreted as an epidemiological indicator of detectable viraemia rather than a diagnosis of confirmed virological failure. In particular, low‐level HIV RNA measurements may represent transient viral blips, persistent low‐level viraemia, or differences in assay sensitivity. Furthermore, HIV RNA was not considered a direct measure of medication adherence because detectable viraemia can arise through several mechanisms, including treatment interruption, drug resistance, drug interactions, and clinical factors [15, 18].

### 2.9. Statistical Data Analysis

Data analysis was conducted using IBM SPSS Statistics Version 28. Descriptive statistics were used to summarize participant characteristics, with categorical variables presented as frequencies and percentages. Associations between categorical variables were assessed using the chi‐square test, and Fisher′s exact test was applied where appropriate for small cell counts [31].

Univariable (crude) and multivariable associations between potential predictors and nonoptimal virological response were assessed using Poisson regression with robust variance. Risk ratios (RRs) were used because the retrospective cohort design established a temporal sequence between cART initiation, assessment of follow‐up HIV RNA, and classification of virological response, allowing the risk of nonoptimal virological response to be compared between exposure groups over the defined follow‐up period [18]. The use of RRs was also considered appropriate because nonoptimal virological response was relatively common in this cohort (41.5%), for which odds ratios may overestimate the magnitude of association and may be less directly interpretable as measures of risk. In contrast, prevalence odds ratios are generally used in cross‐sectional analyses where the outcome is assessed at a single point or period rather than through a defined follow‐up process [32–37]. Potential predictors considered in the regression analysis included sociodemographic characteristics (age, sex, marital status, occupation, year of HIV diagnosis, and imprisonment), behavioral and transmission‐related characteristics (injection drug use and mode of HIV transmission), and clinical characteristics, including reason for HIV testing, WHO clinical stage, hemoglobin level, tuberculosis status, HBsAg positivity, syphilis status, HCV IgG positivity, and cotrimoxazole use. Covariates were measured at baseline. The follow‐up period for each participant was defined as the time from cART initiation to the third eligible HIV RNA measurement, following two consecutive eligible measurements obtained at least 6 months after cART initiation. Variables were retained in the multivariable model according to the regression analysis strategy and statistical significance in the univariable analysis. Adjusted risk ratios (aRRs) with 95% confidence intervals (CIs) were reported. Statistical significance was set at *p* ≤ 0.05.

## 3. Results

### 3.1. Sociodemographic Characteristics of Adults Living With HIV Stratified by Nonoptimal Virological Response in Oman, 1992–2024

Among the 549 adults living with HIV included in the analysis, 321 (58.5%) achieved an optimal virological response (HIV RNA ≤ 20 copies/mL), whereas 228 (41.5%) were classified as having a nonoptimal virological response. Table 1 presents the sociodemographic characteristics of adults living with HIV in Oman (1992–2024), stratified by virological response status based on viral load measurements. Sex (Figure S1), marital status (Figure S2), employment status (Figure S3), and year of diagnosis (Figure S4) were significantly associated with virological response status.

**Table 1:** Sociodemographic characteristics of adults living with HIV stratified by virological response status based on viral load measurements, Oman, 1992–2024.

Characteristics	Total (*n* = 549) *n*(%)	Optimal virological response (*n* = 321) *n* (%)	Nonoptimal virological response (*n* = 228) *n* (%)	*p*
Nationality				0.59
Omani	530 (96.5)	311 (96.9)	219 (96.1)	
Non‐Omani	19 (3.5)	10 (3.1)	9 (3.9)	

Sex				0.006 ^∗^
Male	379 (69.0)	207 (64.5)	172 (75.4)	
Female	170 (31.0)	114 (35.5)	56 (24.6)	

Age (years)				0.81
18–27	146 (26.6)	84 (26.2)	62 (27.2)	
28–37	206 (37.5)	117 (36.4)	89 (39.0)	
38–47	116 (21.1)	72 (22.4)	44 (19.3)	
> 47	81 (14.8)	48 (15.0)	33 (14.5)	

Marital status				0.001 ^∗^
Not disclosed	16 (2.9)	2 (0.6)	14 (6.1)	
Single	194 (35.3)	98 (30.5)	96 (42.1)	
Married	287 (52.3)	188 (58.6)	99 (43.4)	
Divorced	31 (5.6)	20 (6.2)	11 (4.8)	
Widowed	21 (3.8)	13 (4.0)	8 (3.5)	

Employment				0.001 ^∗^
Not disclosed	44 (8.0)	12 (3.7)	32 (14.0)	
Employed	251 (45.7)	170 (53.0)	81 (35.5)	
Unemployed	254 (46.3)	139 (43.3)	115 (50.4)	

Year of diagnosis				0.001 ^∗^
2008 or earlier	142 (25.9)	63 (19.6)	79 (34.6)	
2009–2014	147 (26.8)	73 (22.7)	74 (32.5)	
2015–2019	142 (25.9)	100 (31.2)	42 (18.4)	
2020 or later	118 (21.5)	85 (26.5)	33 (14.5)	

*Note:* Optimal virological response: HIV RNA ≤ 20 copies/mL. Nonoptimal virological response was operationally defined as HIV RNA > 20 copies/mL according to routine clinical monitoring practice. This definition should not be interpreted as confirmed virological failure.

^∗^Significant *p* value ≤ 0.05.

Nonoptimal virological response was more frequent among males than females (75.4% vs. 64.5%; *p* = 0.006). Single individuals were more likely to have a nonoptimal virological response compared with married individuals (42.1% vs. 30.5%; *p* = 0.001), whereas married individuals were more frequently represented in the optimal virological response group (58.6% vs. 43.4%; *p* = 0.001). Employment status was also significantly associated with virological response status (*p* = 0.001), with a higher proportion of employed individuals observed in the optimal virological response group. Year of HIV diagnosis was significantly associated with virological response status (*p* = 0.001). Patients with a nonoptimal virological response were more frequently diagnosed in 2008 or earlier (34.6% vs. 19.6%) and 2009–2014 (32.5% vs. 22.7%), whereas those with an optimal virological response were more commonly diagnosed during 2015–2019 (31.2% vs. 18.4%) and 2020 or later (26.5% vs. 14.5%).

### 3.2. Clinical Characteristics of Adults Living With HIV Stratified by Nonoptimal Virological Response in Oman, 1992–2024

Table 2 summarizes the clinical characteristics of adults living with HIV according to virological response status. Mode of HIV transmission, reason for HIV testing, WHO clinical stage, hemoglobin level, tuberculosis status, HBsAg status, syphilis status, HCV IgG status, and cotrimoxazole use were significantly associated with virological response status. Nondisclosure of HIV transmission mode was more common among patients with nonoptimal virological response than among those with optimal virological response (35.5% vs. 15.6%), whereas sexual transmission was predominant among patients with optimal virological response (75.4% vs. 51.3%; *p* = 0.001). HIV‐related symptoms were the most common reason for HIV testing among patients with nonoptimal virological response (66.7% vs. 47.4%; *p* = 0.001).

**Table 2:** Clinical characteristics of adults living with HIV stratified by virological response status based on HIV RNA viral load in Oman, 1992–2024.

Characteristics	Total (*n* = 549) *n* (%)	Optimal virological response (*n* = 321) *n* (%)	Nonoptimal virological response (*n* = 228) *n* (%)	*p*
Mode of transmission				
Not disclosed	131 (23.9)	50 (15.6)	81 (35.5)	0.001 ^∗^
Sexual	359 (65.4)	242 (75.4)	117 (51.3)	
Vertical	7 (1.3)	5 (1.6)	2 (0.9)	
Parenteral	52 (9.5)	24 (7.5)	28 (12.3)	

Reason for HIV testing				0.001 ^∗^
Not disclosed	6 (1.1)	4 (1.2)	2 (0.9)	
Antenatal care	42 (7.7)	25 (7.8)	17 (7.5)	
Hospital checkup	18 (3.3)	12 (3.7)	6 (2.6)	
HIV contact	103 (18.8)	75 (23.4)	28 (12.3)	
Test request	15 (2.7)	13 (4.0)	2 (0.9)	
HIV symptoms	304 (55.4)	152 (47.4)	152 (66.7)	
Official document/prisoner	61 (11.1)	40 (12.5)	21 (9.2)	

WHO clinical staging				0.001 ^∗^
Stage 1: asymptomatic	213 (38.8)	151 (47.0)	62 (27.2)	
Stage 2: mild symptoms	25 (4.6)	16 (5.0)	9 (3.9)	
Stage 3: advanced symptoms	146 (26.6)	84 (26.2)	62 (27.2)	
Stage 4: severe symptoms (AIDS)	165 (30.1)	70 (21.8)	95 (41.7)	

Comorbidity				0.07
No comorbidity	288 (52.5)	178 (55.5)	110 (48.2)	
Anemia	66 (12.0)	34 (10.6)	32 (14.0)	
Diabetes mellitus	34 (6.2)	20 (6.2)	14 (6.1)	
Hypertension	22 (4.0)	15 (4.7)	7 (3.1)	
Heart disease	15 (2.7)	8 (2.5)	7 (3.1)	
Cancer	51 (9.3)	23 (7.2)	28 (12.3)	
Thyroid disease	9 (1.6)	7 (2.2)	2 (0.9)	
Renal disease	20 (3.6)	11 (3.4)	9 (3.9)	
Psychiatric disorder	22 (4.0)	8 (2.5)	14 (6.1)	
Dyslipidemia	8 (1.5)	7 (2.2)	1 (0.4)	
Respiratory disease	14 (2.6)	10 (3.1)	4 (1.8)	

CART side effect				0.28
Yes	39 (7.1)	26 (8.1)	13 (5.7)	
No	510 (92.9)	295 (91.9)	215 (94.3)	

Hemoglobin				0.001 ^∗^
≤10 g/dL	128 (23.3)	51 (15.9)	77 (33.8)	
>10 g/dL	421 (76.7)	270 (84.1)	151 (66.2)	

Tuberculosis				0.001 ^∗^
Not tested	91 (16.6)	50 (15.6)	41 (18.0)	
Positive	58 (10.6)	21 (6.5)	37 (16.2)	
Negative	400 (72.9)	250 (77.9)	150 (65.8)	

HBsAg				0.020 ^∗^
Not tested	18 (3.3)	5 (1.6)	13 (5.7)	
Positive	32 (5.8)	17 (5.3)	15 (6.6)	
Negative	499 (90.9)	299 (93.1)	200 (87.7)	

Syphilis				0.001 ^∗^
Not done	50 (9.1)	18 (5.6)	32 (14.0)	
Positive	36 (6.6)	26 (8.1)	10 (4.4)	
Negative	463 (84.3)	277 (86.3)	186 (81.6)	

HCV IgG				0.015 ^∗^
Not tested	25 (4.6)	10 (3.1)	15 (6.6)	
Positive	64 (11.7)	30 (9.3)	34 (14.9)	
Negative	460 (83.8)	281 (87.5)	179 (78.5)	

Cotrimoxazole				0.001 ^∗^
Yes	348 (63.4)	177 (55.1)	171 (75.0)	
No	201 (36.6)	144 (44.9)	57 (25.0)	

*Note:* Optimal virological response was defined as HIV RNA ≤ 20 copies/mL (viral suppression). Nonoptimal virological response was operationally defined as HIV RNA > 20 copies/mL according to routine clinical monitoring practice. This definition should not be interpreted as confirmed virological failure.

Abbreviations: CART, combined antiretroviral therapy, HBsAg, hepatitis B surface antigen, HCV IgG, hepatitis C IgG antibody; WHO, World Health Organization.

^∗^Significant *p* value ≤ 0.05.

Regarding disease severity, patients classified as WHO clinical Stage 1 (asymptomatic) were more frequent among those with optimal virological response than among those with nonoptimal virological response (47.0% vs. 27.2%), whereas WHO Stage 4 disease (severe symptoms/AIDS) was more frequent among patients with nonoptimal virological response (41.7% vs. 21.8%; *p* = 0.001). Low hemoglobin levels (≤ 10 g/dL) were more common among patients with nonoptimal virological response (33.8% vs. 15.9%; *p* = 0.001). Tuberculosis positivity was also more common among patients with nonoptimal virological response (16.2% vs. 6.5%; *p* = 0.001; Figure 1). The significant associations observed for HBsAg status and syphilis status were largely attributable to differences in the proportions of patients who were not tested or did not test, rather than differences in positivity. HBsAg positivity was 6.6% among patients with nonoptimal virological response compared with 5.3% among those with optimal virological response, whereas syphilis positivity was 4.4% and 8.1%, respectively. HCV IgG positivity was more frequent among patients with nonoptimal virological response than among those with optimal virological response (14.9% vs. 9.3%; *p* = 0.015). We observed no significant association between cART side effects and virological response status (*p* = 0.280).

**Figure 1:**
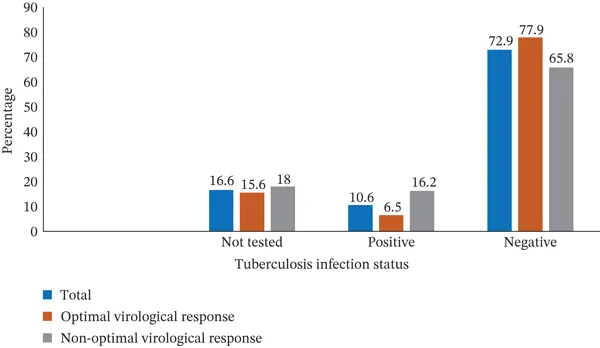
Distribution of tuberculosis status according to virological response status among adults living with HIV in Oman, 1992–2024.

### 3.3. Behavioral Risk Factors Among Adults Living With HIV Stratified by Virological Response Status in Oman, 1992–2024

Table 3 presents a statistically significant association between imprisonment history and virological response status (*p* = 0.04), with a higher proportion of individuals with a history of imprisonment among those with nonoptimal virological response compared with those with optimal virological response (4.8% vs. 1.9%). Smoking status, alcohol consumption, and injection drug use were not significantly associated with virological response status (*p* > 0.05).

**Table 3:** Behavioral risk factors among adults living with HIV stratified by virological response status based on HIV RNA viral load in Oman, 1992–2024.

Characteristics	Total (*n* = 549) *n* (%)	Optimal virological response (*n* = 321) *n* (%)	Nonoptimal virological response (*n* = 228) *n* (%)	*p*
Smoking status				0.11
Yes	110 (20.0)	57 (17.8)	53 (23.2)	
No	439 (80.0)	264 (82.2)	175 (76.8)	
Alcohol consumption				0.07
Yes	92 (16.8)	46 (14.3)	46 (20.2)	
No	457 (83.2)	275 (85.7)	182 (79.8)	
Injection drug use (IDU)				0.11
Yes	35 (6.4)	16 (5.0)	19 (8.3)	
No	514 (93.6)	305 (95.0)	209 (91.7)	
Imprisonment				0.04 ^∗^
Yes	17 (3.1)	6 (1.9)	11 (4.8)	
No	532 (96.9)	315 (98.1)	217 (95.2)	

*Note:* Optimal virological response: HIV RNA ≤ 20 copies/mL (viral suppression). Nonoptimal virological response was operationally defined as HIV RNA > 20 copies/mL according to routine clinical monitoring practice. This definition should not be interpreted as confirmed virological failure.

^∗^Significant *p* value ≤ 0.05.

### 3.4. Multivariable Poisson Regression Analysis of Factors Associated With Nonoptimal Virological Response Among Adults Living With HIV in Oman, 1992–2024

Table 4 presents the multivariable Poisson regression model with robust variance, identifying factors independently associated with nonoptimal virological response among adults living with HIV in Oman (1992–2024). Model estimates are presented as aRRs with 95% CIs. Earlier year of HIV diagnosis was significantly associated with nonoptimal virological response. Patients diagnosed in 2008 or earlier (aRR: 2.166; 95% CI: 1.160–4.043; *p* = 0.015) and those diagnosed between 2009 and 2014 (aRR: 2.273; 95% CI: 1.258–4.107; *p* = 0.007) had approximately twice the risk of non‐optimal virological response compared with patients diagnosed in 2020 or later. Male patients also had a significantly higher risk of nonoptimal virological response compared with (aRR: 2.154; 95% CI: 1.172–3.960; *p* = 0.014). Among clinical characteristics, WHO clinical Stage 3 (advanced symptoms) was independently associated with higher risk of nonoptimal virological response compared with WHO Stage 4 (severe symptoms/AIDS) (aRR: 0.592; 95% CI: 0.349–1.005; *p* = 0.052). In addition, hemoglobin ≤ 10 g/dL was significantly associated with increased risk of nonoptimal virological (aRR: 1.676; 95% CI: 1.020–2.753; *p* = 0.042). Antenatal care (ANC) as the reason for HIV testing was associated with increased risk of nonoptimal virological response (aRR: 3.662; 95% CI: 1.263–10.618; *p* = 0.017). However, this finding should be interpreted cautiously because of the small number of patients in this subgroup, which may affect the precision and stability of the estimate. Other evaluated factors, including marital status, employment status, mode of HIV transmission, HBsAg positivity, syphilis status, tuberculosis, HCV IgG positivity, and imprisonment history, were not significantly associated with nonoptimal virological response after adjustment.

**Table 4:** Multivariable Poisson regression analysis of factors associated with nonoptimal virological response (HIV RNA > 20 copies/mL) among adults living with HIV in Oman, 1992–2024.

Variable	Crude model		*p*	Adjusted model		*p*
	RR	95% CI		aRR	95% CI	
Year of diagnosis						
2008 or earlier	1.99	(1.44, 2.75)	< 0.001	2.166	(1.160, 4.043)	0.015 ^∗^
2009–2014	1.80	(1.29, 2.51)	< 0.001	2.273	(1.258, 4.107)	0.007 ^∗^
2015–2019	1.06	(0.72, 1.55)	0.776	1.156	(0.636, 2.101)	0.634
2020 or later	1.00 ^∗∗^	—		1.00	—	

Sex						
Male	1.38	(1.08, 1.75)	0.009	2.154	(1.172, 3.960)	0.014 ^∗^
Female	1.00	—		1.00	—	—

Marital status						
Not disclosed	2.30	(1.29, 4.09)	0.005	1.542	(0.157, 3.486)	0.168
Single	1.30	(0.74, 2.28)	0.363	1.496	(0.511, 4.381)	0.462
Married	0.91	(0.51, 1.60)	0.732	0.906	(0.329, 2.491)	0.848
Divorced	0.93	(0.45, 1.92)	0.847	0.691	(0.192, 2.490)	0.572
Widowed	1.00			1.00	—	—

Employment						
Not disclosed	1.61	(1.28, 2.01)	0.001	1.786	(0.699, 4.566)	0.226
Employed	0.71	(0.57, 0.89)	0.003	0.649	(0.404, 1.041)	0.073
Unemployed	1.00	—	—	1.00	—	—

Mode of transmission						
Not disclosed	1.15	(0.86, 1.53)	0.342	1.316	(0.529, 3.275)	0.555
Sexual	0.61	(0.45, 0.81)	< 0.001	0.762	(0.322, 1.805)	0.537
Vertical	0.53	(0.16, 1.76)	0.300	0.479	(0.071, 3.226)	0.450
Parenteral	1.00	—	—	1.00	—	—

Reason for testing						
Not disclosed	0.97	(0.30, 3.16)	0.95716	0.003	(< 0.001, 1.807)	0.075
Antenatal care	1.18	(0.71, 1.95)	0.529	3.662	(1.263, 10.618)	0.017 ^∗^
Hospital checkup	0.97	(0.46, 2.03)	0.932	1.823	(0.511, 6.499)	0.354
HIV contact	0.79	(0.49, 1.26)	0.324	1.365	(0.576, 3.233)	0.480
Test request	0.39	(0.49, 1.26)	0.324	0.601	(0.108, 3.354)	0.561
HIV symptoms	1.45	(1.01, 2.09)	0.045	1.234	(0.588, 2.588)	0.578
Official document	1.00	—	—	1.00	—	—

WHO clinical staging						
Stage 1: asymptomatic	0.51	(0.39, 0.65)	< 0.001	0.836	(0.432, 1.616)	0.594
Stage 2: mild symptoms	0.63	(0.36, 1.07)	0.088	0.580	(0.206, 1.632)	0.303
Stage 3: advanced symptoms	0.74	(0.59, 0.93)	0.009	0.592	(0.349, 1.005)	0.052
Stage 4: severe symptoms (AIDS)	1.00	—	—	1.00	—	—

Cotrimoxazole						
Yes	1.73	(1.36, 2.21)	0.001	1.512	(0.927, 2.465)	0.098
No	1.00	—	—	1.00	—	—

Tuberculosis						
Not tested	1.20	(0.93, 1.56)	0.166	0.723	(0.402, 1.301)	0.279
Yes	1.70	(1.35, 2.14)	0.001	1.771	(0.912, 3.437)	0.091
No	1.00	—	—	1.00	—	—

HBsAg						
Not tested	1.80	(1.35, 2.14)	0.001	2.197	(0.390, 12.381)	0.372
Yes	1.17	(0.80, 1.72)	0.424	1.111	(0.497, 2.483)	0.798
No	1.00	—	—	1.00	—	—

Syphilis						
Not tested	1.59	(1.26, 2.02)	0.001	2.012	(0.909, 4.453)	0.085
Yes	0.69	(0.40, 1.18)	0.179	0.762	(0.323, 1.796)	0.534
No	1.00	—	—	1.00	—	—

HCV IgG						
Not tested	1.54	(1.10, 2.17)	0.013	0.911	(0.235, 3.534)	0.892
Yes	1.37	(1.06, 1.77)	0.018	0.776	(0.363, 1.657)	0.512
No	1.00	—	—	1.00	—	—

Imprisonment						
Yes	1.58	(0.99, 2,51)	0.056	0.753	(0.253, 2.239)	0.610
No	1.00	—	—	1.00	—	—

Hemoglobin						
≤ 10 g/dL	1.68	(1.39, 2.03)	0.012	1.676	(1.020, 2.753)	0.042 ^∗^
> 10 g/dL	1.00	—	—	1.00	—	—

*Note:* Optimal virological response was defined as HIV RNA ≤ 20 copies/mL and nonoptimal virological response as HIV RNA > 20 copies/mL.

Abbreviations: aRR, adjusted risk ratio; RR, crude risk ratio; CI, confidence interval; HBsAg, hepatitis B surface antigen; HCV IgG, hepatitis C IgG antibody; WHO, World Health Organization.

^∗^Significant *p* value ≤ 0.05.

^∗∗^Reference category (1.00).

## 4. Discussion

This retrospective cohort analysis examined factors associated with detectable HIV RNA among adults receiving cART at Oman′s national tertiary HIV referral center over 32 years. Using the predefined study threshold of HIV RNA > 20 copies/mL, 41.5% of participants were classified as having a nonoptimal virological response. After adjustment for potential confounding variables, year of HIV diagnosis, sex, WHO clinical stage, antenatal HIV testing, and hemoglobin concentration were independently associated with the outcome.

The interpretation of the outcome requires consideration because the study covered a prolonged period during which HIV laboratory technologies and treatment practices changed substantially. The threshold of > 20 copies/mL was selected according to the laboratory and clinical monitoring framework used at the Royal Hospital. However, a detectable HIV RNA result at this level does not necessarily indicate confirmed virological failure. Depending on the clinical context, low‐level viraemia may represent a transient viral blip, persistent low‐level viraemia, treatment interruption, drug resistance, or variation in assay performance. Accordingly, the current findings should be interpreted as associations with detectable viraemia according to the study definition rather than as evidence of treatment failure.

The current study shares the underlying cohort with our previous publication examining late HIV diagnosis [22]. However, the two analyses address distinct outcomes. The previous investigation focused on factors associated with late diagnosis at the time HIV infection was identified, whereas the current analysis evaluates virological response following cART initiation. The distinction is important because late diagnosis and subsequent virological response represent different stages of the HIV care continuum.

Our study found that male patients had a higher risk of nonoptimal virological response compared with female patients. This finding is consistent with previous studies reporting sex differences in virological outcomes among PLWH. In a large retrospective cohort study from China, women were more likely than men to achieve virological suppression at both 12 and 48 months after ART initiation [38]. Similarly, a longitudinal study from France found that women achieved viral suppression more rapidly than men following ART initiation [39]. These findings provide direct evidence of sex differences in virological outcomes and are broadly consistent with the higher risk of nonoptimal virological response observed among men in our study. However, findings across settings are not uniform. In the Swiss HIV Cohort Study, women were initially less likely than men to achieve virological suppression at 1 and 2 years, although this difference was no longer evident after adjustment for sociodemographic and HIV‐related factors [40]. Evidence from adherence studies may provide additional, but indirect, context. Piegas et al. [41] reported sex‐related differences in ART adherence, whereas Puskas et al. [42] found that 77.1% of men achieved optimal adherence compared with 57.0% of women. However, because adherence was not directly measured in our study, these findings should be interpreted as possible explanatory mechanisms rather than direct comparisons with our virological outcome. Overall, the inconsistent findings across studies may reflect differences in healthcare access, social and cultural context, treatment characteristics, adherence measurement, and population composition. Further research using direct measures of adherence and longitudinal viral‐load outcomes is needed to clarify the factors contributing to sex differences in virological response among PLWH in Oman. Patients diagnosed in 2008 or earlier and 2009–2014 had a higher risk of nonoptimal virological response than those diagnosed in 2020 or later. This association may partly reflect differences across treatment eras, as virological suppression has improved over time with advances in antiretroviral treatment and HIV care [43]. In Oman, viral suppression among people receiving ART increased from 75.7% in 2015 to 87.5% in 2018, alongside improvements in ART coverage and retention in care [2, 3]. The observed association may also reflect differences in cumulative treatment exposure, as individuals diagnosed earlier may have had a longer duration of ART and greater exposure to earlier treatment regimens. In addition, changes in HIV RNA assay technology and analytical sensitivity over the study period may have influenced the detection of low‐level viraemia, with differences in assay performance reported at low HIV RNA concentrations [44]. Therefore, the association between year of HIV diagnosis and nonoptimal virological response should be interpreted in the context of changes in treatment regimens, duration of ART exposure, HIV care systems, and laboratory methods over the study period. This study found that WHO clinical stage was associated with nonoptimal virological response. Patients classified as WHO Stage 3 (advanced symptoms) had a lower risk of nonoptimal virological response compared with those classified as WHO Stage 4 (severe symptoms/AIDS). This finding is inconsistent with previous studies reporting poorer ART‐related outcomes among patients with more advanced WHO clinical stages. A study conducted in Ethiopia found that patients with baseline WHO Stage 1 were significantly more likely to achieve optimal ART adherence compared with those in Stage 4 [45]. Similarly, a retrospective cohort study conducted in northeast Ethiopia reported that patients in WHO Stages 2, 3, and 4 were significantly less likely to adhere to treatment compared with those in Stage 1 [46]. As HIV progresses to advanced stages, patients may experience opportunistic infections and systemic complications resulting from immunosuppression, which may increase treatment burden and negatively affect adherence and long‐term virological outcomes [47]. The difference between our findings and previous reports may reflect variations in study populations, clinical settings, treatment access, and measurement approaches. Further studies are needed to better understand the relationship between WHO clinical stage and virological response.

An increased risk estimate for nonoptimal virological response was observed among patients diagnosed through antenatal HIV screening. However, this subgroup was small, and the association is likely influenced by differences in testing pathways rather than representing a true determinant of virological response. ANC screening typically identifies women tested through preventive or routine maternal healthcare services rather than because of HIV‐related symptoms, which may influence the timing of diagnosis and subsequent treatment pathways. Therefore, this association should be interpreted cautiously and should not be considered causal. Relevant literature indicates that HIV treatment outcomes during pregnancy can vary across settings. UNICEF reported substantial global variation in cART coverage among pregnant and breastfeeding women, particularly in the MENA region [1]. Previous studies have identified several challenges affecting HIV treatment adherence during pregnancy, including limited knowledge regarding prevention of mother‐to‐child transmission [48], emotional distress surrounding a new diagnosis [49], and concerns related to fetal safety or social support [50]. However, these studies were conducted in different epidemiological and healthcare contexts from Oman, and the number of patients diagnosed through ANC in our cohort was limited. Therefore, these findings should not be interpreted as evidence of causation in the current study, and larger local studies are required to better understand this subgroup.

In this study, a hemoglobin level of ≤ 10 g/dL was significantly associated with an increased risk of nonoptimal virological response, suggesting that anemia may represent an important clinical marker associated with poorer HIV treatment outcomes [51]. A study conducted in North America showed that the prevalence of moderate‐to‐severe anemia was higher among PLWH who were not taking cART as prescribed [52]. In contrast, a study conducted in Ethiopia found no significant association between anemia and cART adherence [53]. Anemia among PLWH may reflect underlying disease burden, nutritional deficiencies, opportunistic infections, or other clinical complications, which may negatively affect overall health status and treatment outcomes. Patients with HIV‐associated anemia may experience slower clinical recovery and poorer responses to infection management despite receiving cART [54]. Therefore, monitoring hemoglobin levels and addressing anemia‐related conditions may contribute to optimizing long‐term virological outcomes among PLWH.

### 4.1. Limitations

Several limitations should be considered when interpreting these findings.

First, the retrospective study design limited the availability and consistency of information on several factors that may influence virological response. Variables such as HIV‐related stigma, mental health, treatment literacy, educational attainment, socioeconomic circumstances, social support, and patient‐reported barriers to treatment were not consistently documented in the electronic medical records.

Second, direct measures of medication adherence were unavailable. HIV RNA was therefore used as an objective laboratory measure of virological response; however, detectable viraemia cannot be attributed solely to suboptimal adherence. Treatment interruption, antiretroviral resistance, drug interactions, intercurrent illness, and other clinical factors may also contribute to detectable HIV RNA.

Third, the study covered a 32‐year period during which antiretroviral regimens, viral load assays, treatment guidelines, and clinical monitoring practices changed substantially. Although period of HIV diagnosis was included in the analysis, residual confounding arising from temporal changes in HIV management and clinical practice cannot be excluded.

Fourth, differences in laboratory assay sensitivity across historical periods may have influenced the classification of low‐level viraemia. The use of an HIV RNA threshold of >20 copies/mL may have classified some participants with transient viral blips as having nonoptimal virological response. Therefore, this outcome should not be interpreted as equivalent to confirmed virological failure.

Finally, the study was conducted at a single tertiary referral center. Although the Royal Hospital serves as Oman′s national HIV referral center and receives patients from different regions of the country, the findings may not be directly generalizable to settings with substantially different HIV epidemiology, healthcare systems, or social contexts. Nevertheless, the findings may be relevant and potentially applicable to other countries or healthcare settings with similar HIV epidemiological profiles, healthcare structures, and treatment contexts.

Despite these limitations, the study benefits from a substantial cohort and more than three decades of HIV care data, together with routinely collected laboratory and clinical information from the national tertiary referral center. These features provide valuable longitudinal evidence on factors associated with nonoptimal virological response in the Omani context.

## 5. Conclusion

This retrospective cohort study identified five sociodemographic and clinical factors independently associated with nonoptimal virological response among adults living with HIV receiving cART at Oman′s tertiary HIV referral center: earlier period of HIV diagnosis, male sex, ANC as the reason for HIV testing, WHO clinical Stage 3, and hemoglobin concentration ≤10 g/dL.

These findings suggest that selected demographic and clinical characteristics may help identify individuals who could benefit from closer monitoring and individualized support as part of long‐term HIV care. However, the findings should be interpreted in the context of the retrospective study design, extended observation period, and use of detectable HIV RNA as the operational outcome rather than confirmed virological failure. The association observed among participants diagnosed through ANC should be interpreted cautiously because of the small subgroup size.

Strengthening long‐term engagement in HIV care, supporting treatment continuity, addressing clinical conditions such as anemia, and providing patient‐centered services may contribute to improved virological outcomes. Future prospective and qualitative studies should investigate behavioral, psychosocial, treatment‐related, and healthcare‐system factors that could not be adequately assessed using retrospective medical records and may help identify modifiable barriers to sustained viral suppression in the Omani context.

**Nomenclature**
AIDS: acquired immunodeficiency syndrome
ANC: antenatal care
aRR: adjusted risk ratio/relative risk
cART: combined antiretroviral therapy
CD4: Cluster of differentiation 4
CI: confidence interval
GCC: Gulf Cooperation Council
Hb: hemoglobin
HBsAg: Hepatitis B surface antigen
HCV: Hepatitis C virus
HIV: human immunodeficiency virus
IDU: injection drug use
PLWH: people living with HIV
RR: risk ratio/relative risk
TB: tuberculosis
UNAIDS: Joint United Nations Programme on HIV/AIDS
VL: viral load
WHO: World Health Organization

## Author Contributions

Z.M.A‐Z., F.K., and Y.M.A‐F. formulated the study concept. Z.M.A‐Z., R.A., J.A‐N., U.A‐A., and A.A‐M. collected data. Z.M.A‐Z., Y.M.A‐F., and R.A. cleaned the data. Z.M.A‐Z., F.K., Y.M.A‐F., R.A., I.K.A‐M., and L.S.A‐S. contributed to the literature review and write‐up of the manuscript. Z.M.A‐Z., F.K., Y.M.A‐F., A.S.M., and R.A. conceptualized the methods. Z.M.A‐Z., F.K., Y.M.A‐F, R.A., S.J., A.S.M., S.D.A.S., A.A‐M., Z.A‐B., S.A‐A., and I.K.A‐M. contributed to reviewing the results and writing the manuscript.

## Funding

No funding was received for this manuscript.

## Disclosure

All authors read and approved the final draft of the manuscript.

## Ethics Statement

The study protocol was reviewed and approved by the Research Ethics Committee of the Royal Hospital, Ministry of Health, Oman (MoH/CSR/24/28820), on August 27, 2024. The study was conducted in accordance with the principles of the Declaration of Helsinki. Clinical information for eligible participants who initiated cART between January 1, 1992, and December 31, 2024, was retrospectively obtained from the Royal Hospital Shifaa3+ electronic medical record system. Personal identifiers were removed before analysis, and access to the anonymized dataset was restricted to the research team. The ethics committee waived the requirement for written informed consent because the study involved retrospective analysis of routinely collected deidentified clinical information.

## Conflicts of Interest

The authors declare no conflicts of interest.

## Supporting Information

Additional supporting information can be found online in the Supporting Information section.

## Supporting information


**Supporting Information 1** Figure S1: Sex distribution stratified by virological response status among adults living with HIV in Oman, 1992–2024. Figure S2: Marital status distribution stratified by virological response status among adults living with HIV in Oman, 1992–2024. Figure S3: Distribution of employment status according to virological response status among adults living with HIV in Oman, 1992–2024. Figure S4: Distribution of year of HIV diagnosis according to virological response status among adults living with HIV in Oman, 1992–2024.

**Supporting Information 2** File S1: STROBE statement checklist of items that should be included in reports of observational studies.

## Data Availability

Data are available on request through direct contact with Dr. Faryal Khamis, Senior Consultant, Department of Infectious Diseases, Royal Hospital, Ministry of Health, Oman (email: khami001@gmail.com).
