# Supplementary material for: Nonoptimal Virological Response Among Adults Living With HIV After Initiation of Combined Antiretroviral Therapy at a Tertiary Hospital in Oman: A Retrospective Cohort Study, 1992–2024
Source: ScientificWorldJournal. 2026 Oct 21;2026:8552128. doi: 10.1155/tswj/8552128 (PMC13685659; doi:10.1155/tswj/8552128)
Supplement: Supplementary file 1 [file TSWJ-2026-8552128-s001.docx]

**Supplementary file (Figures 1-4)**

Percentage

| Sex | Male | Female |
| --- | --- | --- |
| Total (n=549) | 69 | 31 |
| Optimal virological response (n=321) | 64.5 | 35.5 |
| Non-optimal virological response (n=228) | 75.4 | 24.6 |

**Figure 1. Sex distribution stratified by virological response status among adults living with HIV in Oman, 1992–2024.**

Percentage

| Marital Status | Optimal virological response | Non-optimal virological response |
| --- | --- | --- |
| Not Disclosed | 0.6 | 6.1 |
| Single | 30.5 | 42.1 |
| Married | 58.6 | 43.4 |
| Divorced | 6.2 | 4.8 |
| Widowed | 4 | 3.5 |

**Figure 2. Marital status distribution stratified by virological response status among adults living with HIV in Oman, 1992–2024.**

Percentage

| Employment Status | Optimal virological response | Non-optimal virological response |
| --- | --- | --- |
| Not disclosed | 3.7 | 14 |
| Employed | 53 | 35.5 |
| Unemployed | 43.3 | 50.4 |

**Figure 3. Distribution of employment status according to virological response status among adults living with HIV in Oman, 1992–2024.**

Percentage

| Year of Diagnosis | Optimal virological response | Non-optimal virological response |
| --- | --- | --- |
| 2008 or earlier | 19.6 | 34.6 |
| 2009 to 2014 | 22.7 | 32.5 |
| 2015 to 2019 | 31.2 | 18.4 |
| 2020 or later | 26.5 | 14.5 |

**Figure 4. Distribution of year of HIV diagnosis according to virological response status among adults living with HIV in Oman, 1992–2024.**
